# Autochthonous Bacterial Isolates Successfully Stimulate *In vitro* Peripheral Blood Leukocytes of the European Sea Bass (*Dicentrarchus labrax*)

**DOI:** 10.3389/fmicb.2016.01244

**Published:** 2016-08-08

**Authors:** Ivona Mladineo, Ivana Bušelić, Jerko Hrabar, Ivana Radonić, Anamarija Vrbatović, Slaven Jozić, Željka Trumbić

**Affiliations:** ^1^Institute of Oceanography and FisheriesSplit, Croatia; ^2^Department of Marine Studies, University of SplitSplit, Croatia

**Keywords:** autochthonous bacteria, peripheral blood leukocytes, probiotic, sea bass

## Abstract

Commercially available probiotics are routinely administered as feed supplements in aquaculture important species. Among them, the European sea bass (*Dicentrarchus labrax*) is the most widely reared fish in the Mediterranean, whose rearing systems are highly variable between countries, affecting at some level the sustainability of production. After random isolation of autochthonous gut bacteria of the sea bass, their identification and pathogenicity testing, we have selected three potentially probiotic isolates; *Pseudoalteromonas* sp., *Alteromonas* sp., and *Enterovibrio coralii*. Selected isolates were tested and their immunostimulative efficiency was compared with a commercially available *Lactobacillus casei* isolate, inferring inflammatory, apoptotic and anti-pathogen response of sea bass’ peripheral blood leukocytes. Phagocytic activity, respiratory burst, and expression of lysozyme, Mx protein, caspase 3, TNF-α, IL-10 genes was measured 1, 3, 5, and 12 h post-stimulation by four bacterial isolates to evaluate early kinetics of the responses. Best immunostimulative properties were observed in *Pseudoalteromonas*-stimulated leukocytes, followed by *Alteromonas* sp. and *L. casei*, while *Enterovibrio coralii* failed to induce significant stimulation. Based on such *in vitro* assay intestinal autochthonous bacterial isolates showed to have better immunostimulative effect in sea bass compared to aquaculture-widely used *L. casei*, and further steps need to engage tank and field feeding trials to evaluate long-term prophylactic suitability of the chosen isolates. A panel of biomarkers that represent pro-/anti-inflammatory, pro-/anti-apoptotic, and anti-bacteria/viral responses of the fish should be taken into consideration when evaluating the usefulness of the potential probiotic in aquaculture.

## Introduction

Representing the first marine non-salmonid species commercially cultured in Europe, the European sea bass (*Dicentrarchus labrax*) is known today as the most important aquaculture fish in the Mediterranean, reaching production over 260.000 tons in 2013 (FAO, 2014). Greece, Turkey, Italy, Spain, Croatia, and Egypt are the largest producers, although rearing systems can vary from more extensive as coastal lagoons, to the intensive, highly productive inland recirculation systems or net cages. Rearing conditions of each of these systems, primarily water and food traits, fish density and its genetic background, always represent a trade-off between the ultimate health status of the fish and its growth rate. Therefore, specific rearing systems in particular geographic regions can express somewhat different etiological and epidemiological frame for the fish welfare, important to consider during the planning of zooprophylactic measures.

Among the later, probiotics stand as beneficial and easy to use feed additives composed of live microorganisms that exhibit an antimicrobial effect through modifying the intestinal microbiota, secreting antibacterial substances (bacteriocins and organic acids), competing with pathogens to prevent their adhesion to the intestine, competing for nutrients necessary for pathogen survival, and producing an antitoxin effect. Additionally, they are also capable of modulating the host immune system, regulating allergic response of the body, and reducing proliferation of cancer in mammals (Schrezenmeir and de Vrese, 2001). Martínez Cruz et al. (2012) gave a detailed review of probiotic use and effects in aquaculture organisms, while studies focusing specifically on their role in stimulating immune response were undertaken for common (*Cyprinus carpio*; Wang et al., 2010; Zhang et al., 2011; Chi et al., 2014) and grass carp (*Ctenopharyngodon idella*; Wu et al., 2012), orange-spotted grouper (*Epinephelus coioides;* Cheng et al., 2009; Sun et al., 2010), hybrid tilapia (*Oreochromis niloticus* × *O. aureus*; He et al., 2009), eel (*Anguilla japonica*; Lee et al., 2013), cobia (*Rachycentron canadum*; Geng et al., 2011), rainbow trout (*Oncorhynchus mykiss*; Newaj-Fyzul et al., 2007; Sharifuzzaman et al., 2011) and olive flounder (*Paralichthys olivaceus*; Harikrishnan et al., 2010). Different bacteria have been also tested as probiotics in the Mediterranean aquaculture for sea bass and sea bream (*Sparus aurata*); *Lactobacillus fructivorans, L. plantarum*, and *L. delbrueckii* (Carnevali et al., 2004, 2006; Silvi et al., 2008), *Enterococcus* spp. (Bourouni et al., 2012), *Bacillus subtilis* (Cerezuela et al., 2012b, 2013), *Tetraselmis chuii* and *Phaeodactylum tricornutum* (Cerezuela et al., 2012a). Moreover, bacteria of autochthonous origin were proposed to have a greater capability to compete with resident pathogens, and were also more prone to dominate and persist over other potentially pathogenic microbes (Nayak, 2010).

While studies focusing on the probiotic potential of autochthonous bacteria in fish mostly encompass *in vivo* feeding trials, less information is available about *in vitro* stimulation of peripheral blood leukocytes (PBL), leaving out important information about early kinetics of immune response to probiotics. Therefore, the aim of this study was to (i) isolate autochthonous gut bacteria of reared sea bass and characterize them biochemically and molecularly; (ii) test the pathogenic potential of at least three selected isolates inferred by their hemolytic activity, resistance to antimicrobial compounds and *in vivo* pathogenicity test; (iii) test the innate immune response [respiratory burst (RB), phagocytic activity (PA), and expression of lysozyme, Mx protein, caspase 3, TNF-α, IL-10 genes] of PBLs to three selected isolates and one commercially available (*L. casei*).

## Materials and Methods

### Fish

Three-year old European sea bass (*Dicentrarchus labrax*; *N* = 50) weighting 353.79 g ± 83.40 (mean ± SD) and 33.60 cm ± 5.45 (mean ± SD) in length, were transported from a nearby farm to the experimental hatchery of the Institute of Oceanography and Fisheries, and kept in concrete, flow-through tanks (12 m^3^) for an acclimation period of 30 days, fed their commercial diet. Water parameters were measured every day during the course of experiment; salinity (‰) and temperature (°C) by a probe, oxygen quantity (ml/L) and saturation (%) by modified Winkler method (Carpenter, 1965), and ammonia (μmol/L) following Deggobis (1973; data not shown).

### Bacterial Isolation and Identification

Immediately upon arrival, five fish were euthanized by an overdose of MS222, their abdominal surface was disinfected by 70% ethyl-alcohol, intestine from pyloric to anal region was dissected, opened, and washed with sterile PBS.

Intestine tracts of each fish were collected, pooled, weighted and ninefold (w/v) diluted using sterile 0.85% physiological saline. After 60 s homogenization in sterile blender, homogenate was serially diluted using a ninefold sterile (v/v) physiological saline. Aliquots (200 μl) of each dilution were separately spread on 160 mm diameter Marine agar 2216 plates (Difco) and incubated at 25°C for 72 h. In order to obtain potential diversity, plate with 100–200 clearly separated bacterial colonies was chosen. All colonies were counted and characterized based on colony morphology and gram staining. After testing the purity, biochemical identification was performed following standard taxonomic schemes (Shewan et al., 1960; Oliver, 1982; Surajit et al., 2007).

At the same time, DNA was isolated from selected colonies (*N* = 70) using Quick DNA small tissue and blood kit and amplified at 16s rRNA locus, using previously published primers and parameters (Wilson et al., 1990) (Supplementary Table S1). PCR products were sent to Macrogen, Amsterdam (the Netherlands) for commercial sequencing. Obtained sequences were aligned in MEGA 6.0 (Nei, 1987), and analyzed after trimming of primer annealing sites using BLAST tool^1^ to search for identity in the GenBank and RBD (Ribosomal Data Base Project II)^2^. Generated sequences were added to GenBank (accession numbers KX356388 – KX356457). Taxonomic identification was determined based on similarity of >99%. Stock cultures of identified isolates were stored in 20% glycerol at -80°C.

### Resistance to Antimicrobial Compounds

In order to prevent multiplication during *in vitro* assay of PBLs immunostimulation, bacterial cultures were tested for penicillin/streptomycin sensitivity. Disk diffusion method was performed by using 6 mm disks previously saturated with 10 μL of penicillin/streptomycin solution (10,000 U/ml Penicillin; 10,000 μg/ml Streptomycin).

### Pathogenicity Testing

Standard test for hemolytic activity of selected bacterial isolates was used to assess *in vitro* potential pathogenicity. Briefly, bacteria were grown on sheep blood agar plates and the hemolysis was determined according to Luis-Villaseñor et al. (2011) as: α-hemolysis that leads to slight destruction of erythrocytes with a green circle around the bacterial colonies, β-hemolysis that causes a clean hemolysis zone around colonies, and γ-hemolysis that induces no change on the agar plates around the colonies.

Based on identification results (Supplementary Table S1), three γ-hemolytic autochthonous bacterial isolates were selected for further testing of probiotic-stimulated immunity; *Enterovibrio coralii*, as the most prevalent, and *Alteromonas* sp. and *Pseudoalteromonas* sp. as the rarest isolates but previously reported as probiotic (Liu, 2016). In addition to these, commercially available *L. casei* was used in immunity assays as a positive control, because this bacteria often produces bacteriocins and other chemical compounds that may inhibit the growth of pathogen bacteria and is therefore considered a model probiotic (Gildberg et al., 1997). For all downstream experiments, four bacterial isolates were used.

In addition, each of bacterial isolates was re-cultured and diluted to obtain two concentrations; 10^5^ CFU/ml (low) and 10^8^ CFU/ml (high) that were inoculated intraperitoneally in 10 fish (1 ml/per fish; *N* = 80). Sterile saline (0.85%) was inoculated instead of isolates in the control group (1 ml/per fish; *N* = 40). Survival and potential clinical symptoms were monitored through 30 days, during which fish were regularly fed. No clinical symptoms or mortalities were recorded for any of four selected isolates. For downstream experiments, concentration of 10^8^ CFU/ml of bacteria was used.

The concentration of bacteria in suspensions was determined by the direct method of epifluorescence microscopy (Porter and Feig, 1980) and confirmed by plating on Marine agar (*E. coralii, Alteromonas* sp., and *Pseudoalteromonas* sp.) and MRS agar (*L. casei*).

### *In vitro* Assay of Peripheral Blood Leukocytes (PBL) Immunostimulation by Four Bacterial Isolates

Blood of ten MS222-anesthetized fish was drawn aseptically from a caudal vein in 5 ml syringes previously washed by sterile EDTA to prevent coagulation. PBL were prepared as previously described (Lepen Pleić et al., 2015) using 51% iso-osmotic Percoll (Gibco) solution. Briefly, blood was diluted 1:5 in Leibovitz’s medium (L-15), supplemented with 10 units/ml of heparin, 2% fetal calf serum (FCS; Invitrogen Life Technologies), 1% penicillin/streptomycin (P/S; 10,000 U/ml Penicillin G sodium; 10000 mg/ml Streptomycin sulfate) and PBL were separated by centrifugation in Percoll solution, at 2000 × *g* for 20 min without a break. Cells were washed twice in L-15 medium supplemented with 15% FCS and 1% P/S, and visualized in Neubauer hemocytometer following trypan blue staining to ensure viability >95% and final concentration of 10^7^ cells/ml. PBL were seeded in 6- and 96-well plates

### Quantification of Target Gene Expression in PBL Seeded into 6-well Plates

Settled PBL were stimulated with four bacterial isolates by adding them in culture media at concentration of 10^8^ CFU/ml for 1, 3, 5, and 12 h in triplicates. In control wells, only culture media was added. Treatments were terminated by resuspending cells in 1 ml of TriReagent (Ambion) used for PBL total RNA extraction, as per manufacturer’s protocol. RNA was dissolved in 20–40 μl of RNase/DNase free water (Sigma–Aldrich) and quantified using Genova Nano Micro-volume Spectrophotometer (Janway). RNA was firstly treated by 1 unit/μl RNase free DNase I (Fermentas Life Sciences), and afterward cDNA was synthesized from 2 μg of total RNA using High Capacity cDNA reverse Transcription kit (Life Technologies), following the manufacturer’s instructions.

The expression of target genes: lysozyme g (*lys*), Mx protein (*Mx*), caspase 3 (*casp3*), cytokines interleukin 10 (*IL-10*), and tumor necrosis factor α (*TNF*-α), and reference genes 18s ribosomal RNA (*18s rRNA*), ß-actin (*bact*), elongation factor I α (*EF*) was quantified by real-time PCR using LightCycler 480 SYBR Green I Master (Roche) in a LightCycler 480 System set for Relative quantification experiment. Template cDNA was diluted 1:5 in TE buffer (pH 8.0) and each sample was run in duplicate. Primers used for amplification of selected genes and their real-time PCR cycling protocols were as described previously (Buonocore et al., 2014; El Aamri et al., 2015) or designed by Primer3 platform using GenBank sequences as base (AJ537421 for *bact*, AJ866727 for *EF*; Supplementary Table S2). Transcript levels of target genes were calculated using the LightCycler 480 System integrated software. Expression levels of sea bass PBL target genes cDNAs were normalized to the two reference genes, after exclusion of *bact*, characterized by the least stable expression as shown by BestKeeper (Pfaﬄ et al., 2004). The relative expression (presented as arbitrary units) was calculated as a ratio of the expression of the target gene and geometric mean of the expression of *18s rRNA* and *EF*, multiplied by 1,000. Fold change of gene expression after PBL stimulation was calculated as the average expression level of stimulated samples divided by that of the time-matched controls. Following logarithmic transformation (base 2), the fold changes (FCs) observed during the experiment were visualized using line and point plots as implemented in ggplot 2 package (Wickham, 2009) for R software (version 3.2.2). Twofold induction was considered as the threshold for biological significance. Furthermore, principal component analyses (applied through prcomp function in R base package) of FC profiles was used to inspect the overall similarities/differences of genes and bacterial isolates. Sample scores and variable loadings on first two principal components were plotted using ggbiplot package (Vu, 2011) for R. Two-dimensional hierarchical clustering and heatmap display were used to group the genes according to their expression profiles and bacterial isolates based on elicited responses, using default setting in gplots package (Warnes et al., 2016) for R software.

Testing the null hypothesis of no differences in expression level of all target genes was performed using the PRIMER 6 and Permanova + B20 package (Clarke and Gorley, 2006; Anderson et al., 2008). The univariate tests in Permanova were based on Euclidean distance measure and the *p*-values set at *p* < 0.05 were obtained by unrestricted permutation of raw data (999) with Monte-Carlo simulation included.

### Phagocytic Activity (PA) in PBL Seeded into 96-well Plates

Phagocytic activity was evaluated following modified method of Ai et al. (2006). Settled PBL were stimulated with four bacterial isolates by adding 100 μl of 10^8^ CFU/ml in culture media for 1, 3, 5, and 12 h in triplicates at 25°C. After double washing of PBL in L-15 to wash out remaining bacteria, 100 μl *Saccharomyces cerevisiae* type II suspension (Sigma; 10^8^ yeast cells/ml) was added to PBL and left for 2 h. In negative control wells, yeast was added in culture media without stimulation with bacterial isolates. Phagocytosis was interrupted by fixing of cell monolayer in methyl-alcohol and PBL were visualized by staining with Giemsa. Suspension of Giemsa stained cells was smeared on the microscopy slide and inspected under highest magnification (Leica DMI 4000 B). At least 100 PBL were enumerated per each slide, and the percentage of cells containing yeast cells was calculated. Difference between PA of PBL stimulated by four bacteria isolates at four time-points was tested using three-way PERMANOVA with bacterial type and time as orthogonal factors, while cell culture was nested within each of those two factors. Significance was set at *p* < 0.05, with *p*-values being obtained using 999 permutations of row data with Monte-Carlo simulation included.

### Respiratory Burst (RB) in PBL Seeded into 96-well Plates

Respiratory burst activity of leucocytes was quantified by measuring the reduction of nitroblue tetrazolium (NBT) according to modified method of Anderson et al. (1998). To settled PBL, firstly 150 μl/well of 0.3% NBT in culture media was added, and then cells were stimulated with four bacterial isolates by adding 10 μl of 10^8^ CFU/ml in culture media for 1, 3, 5, and 12 h in triplicates at 25°C. Control wells included PBL monolayer without bacterial stimulation, while blank control consisted only of KOH/DMSO added to the PBL. Afterward, PBL monolayer was washed twice with L-15, fixed with 100 and 70% methyl-alcohol and left to dry. For dissolving of reduced NBT, 2N KOH and DMSO were added, stirred and ODs were read by a spectrophotometer at 630 nm. Difference between RB of bacteria-stimulated and unstimulated control PBL was tested between time-points and isolates, using three-way PERMANOVA included in Primer, as described above.

### Ethical Statement

This study was approved by the Ethical Committee for the Animal Welfare of the Institute of Oceanography and Fisheries, Split, being carried out in accordance with the recommendations of “Regulations of protection of animals employed in scientific purposes” Act of Republic of Croatia (NN 55/13).

## Results

### Target Genes Expression in Bacteria-Stimulated PBL

**FIGURE 1:**
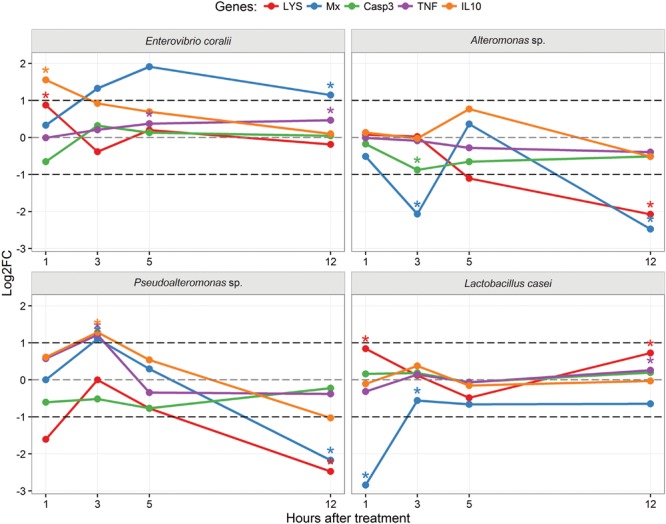
**Log (base 2) transformed fold changes (Log2FC) in gene expression observed during the course of stimulation of sea bass PBLs with different bacterial isolates, in respect to their time-matched controls.** Dark dashed lines depict twofold induction/suppression (1/-1 in log space), considered as the threshold for biological significance. Statistically significant results are marked with ^∗^ (*p* < 0.05).

Bacterial isolates that induced largest differences in expression level (FC) of target genes were identified by Permanova for statistically significant differences, and biologically as at least twofold change (**Supplementary Figure S1**). Since we considered biological significance to be more informative, in the following text we will refer to values that expressed statistical significance at fold change level. *Pseudoalteromonas* sp. showed to be the best probiotic candidate based on *in vitro* assay, significantly downregulating lysozyme (1 and 3 h), upregulating (3 h) then downregulating (12 h) Mx protein, and upregulating TNF*-*α and IL-10 (3 h). *Alteromonas* sp. significantly downregulated lysozyme (5 and 12 h), Mx protein (3 and 12 h), not affecting two cytokines’ expressions. *Enterovibrio coralii* significantly upregulated Mx protein (3–12 h) and IL-10 (1 h), not affecting lysozyme or TNF*-*α expressions. *L. casei* significantly downregulated only Mx protein (1 h), not affecting other targets’ expressions. Log2 fold change expressions of target genes are shown in **Figure 1**. Caspase 3 expression was downregulated by all studied isolates, significantly only in *Alteromonas*-stimulated PBL. Both cytokines responded at 3 h post-stimulation, most markedly in *Pseudoalteromonas* sp.; IL-10 started with stronger expression, while TNF*-*α maintained its levels higher by 12 h. Lysozyme was mainly not affected or downregulated by all isolates, except *L. casei*, but at no biologically significant level.

**FIGURE 2:**
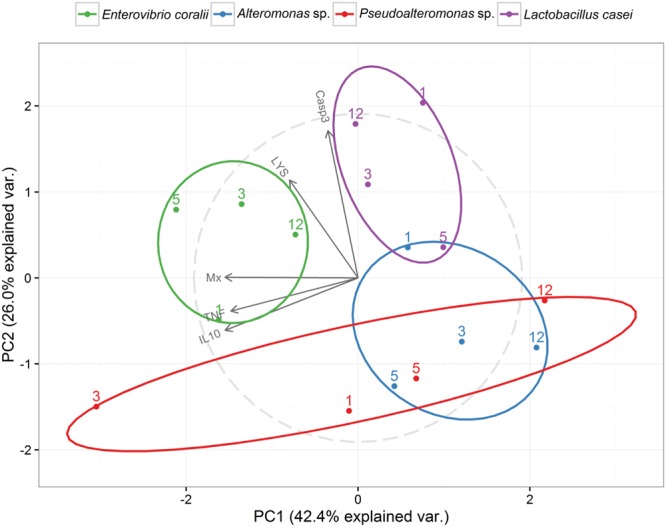
**A biplot of the first two components of principal component analyses performed on FCs of gene expression observed during *in vitro* stimulation of sea bass PBLs with different bacterial isolates.** Relative positions of treatment groups (time-points and isolates) are shown and the variables (genes) depicted as vectors in a correlational circle.

The biplot representation of the results of principal component analyses of gene expression (di)similarities over time-points and bacterial isolates is shown in **Figure 2**. First two principal components explained almost 70% of the variability observed in the data. The ellipses delineate the spread of specific time-points of PBL stimulation with bacterial isolates. *Pseudalteromonas* sp. induced most varying alterations in gene expression that partially overlapped with those of *Alteromonas* sp. (specifically for 5 and 12 h post-stimulation). The other two isolates formed more confined and separate clusters. In respect to genes, the biplot shows congruent patterns of two cytokines, diverging response of Mx protein, driving large part of the variability observed by the first component, and somewhat contrasting patterns of lysozyme and caspase 3. Heatmap representation of hierarchical clustering of gene expression over time-point and isolates is shown in **Supplementary Figure S2**.

### Phagocytic Activity (PA) Induced by Bacterial Isolates in PBL

Highest PA was reached by *Pseudoalteromonas* sp. (3 h), but the steadiest activity that lasted till 12 h post-stimulation was observed in *L. casei*-stimulated cells (**Supplementary Figure S3A**; Supplementary Table S3). All isolates induced statistically different PA at 1 and 3 h, but differences were not observed at 5 and 12 h. Within time-points, *Pseudoalteromonas* sp. and *L. casei* displayed difference between 3 and 12 h, and 1 and 3 h, respectively. In case of *Alteromonas* sp., difference was significant between PA of 1, 3, 5, and 12 h. No difference was observed in PA at four time-points in *E. coralii*-stimulated cells.

### Respiratory Burst (RB) Induced by Bacterial Isolates in PBL

Statistically significant difference between RB in bacteria-stimulated and control PBL was detected in all isolates, except *Alteromonas* sp., mostly showing the highest activity in the first hours post-stimulation (*L. casei* 1–5 h; *E. coralii* 1–12 h; *Pseudoalteromonas* sp. 1–3 h). Consequently, *Alteromonas* sp. was the most different to other three isolates.

*Lactobacillus casei* induced highest RB immediately 1 h post-stimulation compared to other bacterial isolates, but was significantly reduced afterward, reaching the lowest levels at 12 h (**Supplementary Figure S3B**; Supplementary Table S4). *Pseudoalteromonas* sp. increased RB till 5 h, maintaining the highest level at 12 h among studied isolates. *E. coralii* had lower although similar RB pattern to *Pseudoalteromonas* sp., while *Alteromonas* sp. did not induce any significant changes.

## Discussion

Fish intestinal autochthonous bacteria have been suggested as a main source of potential probiotics in aquaculture that, along the rest of gut microbiota, are able to affect the enterocytes architecture and closely relate to physiological functioning of the immune system (Sonnenburg et al., 2006). *In vitro* stimulation of sea bass leukocytes performed here by three autochthonous, potential probiotic, and one commercially available isolate showed that the best immunostimulative effect is induced by autochthonous *Pseudoalteromonas* sp., followed by *Alteromonas* sp. *L. casei* showed not to be highly effective, while a negligible effect has been observed by *E. coralii* stimulation. These results give the basis for development of further feed trials in aquaculture-reared sea bass.

Lysozyme is involved in hydrolization of bacterial cell wall’s peptidoglycan polymer that in Gram-negative bacteria (tested autochthonous bacteria from sea bass gut) is not directly accessible because of their outer membrane, in contrast to that of Gram-positive species (commercially available *L. casei*; Masschalck and Michelis, 2003). In the sea bass, g-type (goose-type) lysozyme basal expression showed to be highest in the gills, then head kidney and PBL (Buonocore et al., 2014), being mostly upregulated after *in vitro* or *in vivo* stimulation with LPS or challenge with bacterial pathogens (Jimenez-Cantizano et al., 2008; Larsen et al., 2009; Buonocore et al., 2014). In contrast, we observed a significant downregulation of *lys* in *Pseudoalteromonas* sp. and *Alteromonas*-stimulated PBL cell that potentially could be attributed to the native character of the isolates, in addition to an innate lysozyme resistance of these bacteria. Namely, lysozyme’s antibacterial activity has been thoroughly studied in pathogens like staphylococci, micrococci, and *Listeria monocytogenes* (Bera et al., 2007), and in lactic acid bacteria, the resistance to lysozyme at 25–35 mg/l was recommended as a criterion for their use in milk industry (Guglielmonti et al., 2007). There are many adaptations in bacteria that enable such resistance; in *Staphylococcus aureus*, peptidoglycans have been altered by *O*-acetylation at the C-6 position of the *N*-acetyl muramic acid impeding hydrolization. It seems that the active site cleft of lysozyme binding with *O*-acetylated peptidoglycan in *S. aureus* hinders sterically, developing a lower structural stability of the lysozyme-peptidoglycan complex (Pushkaran et al., 2015). In other bacteria, cell wall possesses a high degree of cross-linking and a teichoic acid that also contribute to this resistance (Bera et al., 2007). In addition, the lysozyme resistance is governed by two group of genes; those that synergistically block muramidase activity, and those that affect its cationic antimicrobial peptides (Herbert et al., 2007). Although we did not perform testing of our bacterial isolates for lysozyme resistance and are not able to discern underlying mechanisms, downregulation of *lys* transcripts by two isolates is encouraging for their selection as probiotics. Few other exceptions in fish exist when lysozyme was not induced by LPS stimulation or pathogens, both in the Atlantic salmon, but without a clear explanation of mechanisms (Paulsen et al., 2003; Myrnes et al., 2013).

We have observed a significant upregulation of Mx protein immediately post-stimulation followed by downregulation till 12 h, with highest fold change induced by *E. coralii* and *Pseudoalteromonas* sp. Mx proteins belong to dynamin-like large guanosine triphosphatase (GTPases) important for trafficking of intracellular vesicles, organelle homeostasis and mainly anti-RNA viruses activity (Haller et al., 2007) inducible by proinflammatory cytokine interferon-γ (IFN-γ). Mx accumulates in the cytoplasm and nucleus, interacting with endoplasmatic reticulum sub-compartments or nuclear bodies, respectively, where they recognize nucleocapsid-like structures, probably binding to viral polymerase and inhibiting its replication (for review, see Verhelst et al., 2013). In fish, majority of studies have been conducted to assess suppression of aquaculture important viral pathogens by *Mx*; infectious salmon anemia virus (Jensen and Robertsen, 2002; Kibenge et al., 2005) and infectious pancreatic necrosis virus in the Atlantic salmon (Larsen et al., 2004), rhabdoviruses in Japanese flounder *Paralichthys olivaceus* (Caipang et al., 2003), nodavirus in grouper *Epinephelus coioides* (Lin et al., 2006; Chen et al., 2008) and barramundi (Wu et al., 2010), and reovirus in rare minnow *Gobiocypris rarus* (Su et al., 2009). In addition to viruses, *Mx* upregulation has been demonstrated also following injection of pathogenic bacteria *Edwardsiella tarda, Streptococcus iniae*, and *Vibrio* spp. (Acosta et al., 2004; Sun et al., 2011; El Aamri et al., 2015) or their bacterin (Salinas et al., 2004). This along our results that evidenced upregulation of *Mx* by specific probiotics, indicates that Mx role in fish immunity is broadened by its ability to interact with bacterial in addition to viral agents, most likely through triggering of IFN-γ (Zou and Secombes, 2011). Interestingly, p47 GTPase that belongs to the other family of interferon-induced GTPases is known to be inducible by LPS, various intracellular protozoa (*Toxoplasma gondii, Trypanosoma cruzi, Leishmania major*) and bacteria (*Listeria monocytogenes, Salmonella typhimurium, Mycobacterium avium, M. tuberculosis*; Taylor et al., 2004). It still remains to clarify if *Mx* broadened and less specific spectrum in fish is linked to their evolutionary position of immune system compared to that of mammals (Verhelst et al., 2013).

Pro-inflammatory cytokines (TNF-α, IFN-γ, IL-1β) induce antimicrobial functions of immune cells, facilitating clearance of the pathogen, and are balanced by activity of anti-inflammatory cytokines (TGF-β, IL-10) that downregulate inflammatory processes, directing cell functions toward tissue repair mechanisms (Grayfer and Belosevic, 2013). Among others multiple functions, TNF-α promotes the chemotaxis of neutrophils and monocytes/macrophages, enhances their phagocytic capacity, primes ROI and NO responses, chemoattracts fibroblasts and elicits platelet activating factor production (Grayfer and Belosevic, 2013). In contrast, IL-10 is an anti-inflammatory cytokine, inhibiting a broad spectrum of immune responses, including immune cell activation, production of cytokines and chemokines, as well as pathogen resistance, although its function in teleosts is still unclear (Wei et al., 2014). Only stimulation by *Pseudoalteromonas* sp. induced significant but not remarkably high (2.3 FC) expression of TNF-α that after peaking at 3 h post-stimulation decreased. *L. casei* significantly upregulated TNF-α only at 12 h, but below what is considered as biologically important (1.1 FC). IL-10 expression was congruent with that of TNF-α in *Pseudoalteromonas*-stimulated PBL (peaking at 3-h and descending afterward), while *L. casei* did not induce any significant changes in this anti-inflammatory cytokine. Surprisingly, *E. coralii* highly expressed IL-10 immediately 1 h post-stimulation, while its TNF-α expression was not significant during the whole experiment. In general, the interaction of commensal bacteria and probiotics with the surface of antigen-presenting cells *in vitro* results in the downregulation of pro-inflammatory genes that are linked to inflammatory signaling pathways. Subsequently, these bacteria induce a tolerogenic and hyporesponsive immune response in which in particular those genes expressing anti-inflammatory cytokines, are upregulated (Plaza-Diaz et al., 2014). In fish, however, it is difficult to extract a consistent pattern of behavior of pro- and anti-inflammatory cytokines in respect to probiotic stimulation, because there are unlikely two similar designs of study been undertaken. For example, while *L. rhamnosus, Enterococcus faecium*, and *Bacillus subtilis* do upregulate the pro-inflammatory cytokines (IL-1β1 and TGF-β; Panigrahi et al., 2007), it is suggested that expression of IL-1β, IL-8, TNF-α, and TGF-β indicates their potential role in anti-inflammatory response in *Oncorhynchus mykiss* (Kim and Austin, 2006). Giri et al. (2016) used heat-killed whole-cell products (HKWCP) of probiotic *Pseudomonas aeruginosa* VSG2 strain and stimulated the cytokine responses in the head kidney macrophages (HK) of *Labeo rohita*. Authors observed that both TNF-α and IL-10 had significantly higher expression with HKWCP after 2–16 and 2–24 h, respectively, showing a descending kinetics from 2 h onward. Such pattern is similar to what we have observed in PBL.

Expression of a pro-apoptotic factor caspase 3 was downregulated, but statistically significant difference compared to untreated PBL was observed only at 3 h post-stimulation with *Alteromonas* sp. This is congruent with previous studies in fish; in *Labeo rohita*, degree of apoptosis in different tissues was significantly reduced in probiotic-supplemented groups (Mohapatra et al., 2014), in zebrafish fed by diet-supplemented *L. rhamnosus* (Gioacchini et al., 2014) a decrease in the abundance of apoptotic-related genes was observed in the liver. Similarly, *in vitro* testing of extract of *L. delbrueckii* subsp. *lactis* evidenced anti-apoptotic effect in fibroblast-like (SAF-1) and epithelial (EPC) cell lines (Salinas et al., 2007). Extrinsic cell apoptosis is enabled by several receptors, including first apoptosis signal (FAS; also called CD95 or APO-1), tumor necrosis factor receptor 1 (TNFR1), TNF-related apoptosis-inducing ligand (TRAIL) receptor 1 (TRAIL-R1 or DR4) and TRAIL receptor 2 (TRAIL-R2 or DR5). These mediators can be activated by extracellular ligands initiating a protein-protein interaction at cell membranes, activating an intracellular caspase cascade (Altonsy et al., 2010; Circu and Aw, 2010). In men, both pro- and anti-apoptotic effects have been described as beneficial features of probiotics in intestinal pathologies (Muñoz-Atienza, 2015), because pro-apoptotic state can suppress the number of active monocytes and lymphocytes in chronic inflammatory diseases (Chiu et al., 2010) or limit the number of carcinogenic cells in tumors (Chen et al., 2012). In contrast, the anti-apoptotic effect of probiotics in epithelial intestinal cells could reduce the colonic barrier disruption in chemical-induced colitis (Yan and Polk, 2002; Yan et al., 2011). Administration of antibiotics in aquaculture is mainly done via feed, but consequences on the enterocytes have not been studied in depth. In man it has been shown that intestinal mucositis induced by anti-cancer therapy (Prisciandaro et al., 2012), as well as neonatal gastroenteric disbalances experimentally induced in mice (Preidis et al., 2012), can be significantly attenuated by application of probiotics that modulate apoptosis in enterocytes. Namely, changes in caspases 3 and 7 induced by therapeutics or pathogens can be prevented by probiotic factors, inhibiting enterocytes apoptosis and consequent loss of intestinal barrier function. Such cyto-protective effect of probiotics on enterocytes and hepatocytes (Sharma et al., 2011) should be further explored in aquaculture, not solely from the aspect of better post-therapeutic restoring of gut mucosa, but also in context of better nutrient absorption and subsequent increase in growth rate in healthy fish. Taken all together, silencing of apoptosis as observed by *Alteromonas* sp. seems an additional beneficial trait to be sought in autochthonous probiotics.

Finally, some discrepancies were observed when aligning PA and RB within the same probiotic isolate. Kinetics of both processes were congruent up to 5 h, but tended to diverge till 12 h post-stimulation for most isolates. Exceptions where PA was higher in contrast to RB at the end of experiment was observed in *E. coralii*, and vice versa where RB was higher than PA at 12 h was observed in *Alteromonas* sp. PA is mostly stimulated by addition of probiotics in aquaculture feed, while contradictory studies exist in respect to RB tested both *in vivo* and *in vitro* (for review, see Nayak, 2010).

## Conclusion

Based on *in vitro* assay of PBL we concluded that intestinal autochthonous bacterial isolates can elicit better immunostimulative effect in sea bass compared to aquaculture-widely used *Lactobacillus* spp. *Pseudoalteromonas* sp. and *Alteromonas* sp. showed better properties then *E. coralii*, and further steps need to engage tank and field feeding trials to evaluate long-term prophylactic suitability of the chosen isolates. A panel of biomarkers that represent pro-/anti-inflammatory, pro-/anti-apoptotic and anti-bacteria/viral responses of the fish should be taken into consideration when evaluating the usefulness of the potential probiotic in aquaculture.

## Author Contributions

Substantial contribution to the design of the work was given by IM. Acquisition, analysis and interpretation of data was done by all authors (bacterial isolation and identification: SJ, IM, JH, and IR; PBL assay: IM, IB, JH, and AV; qPCR: IB, JH, and ŽT; respiratory burst: IR; phagocytic activity: AV). Drafting the work was done by IM, and all authors contributed in drafting of their area of expertise and revised it critically. All authors have read and approved the final version to be published, and agreed to be accountable for the statements in this work.

## Conflict of Interest Statement

The authors declare that the research was conducted in the absence of any commercial or financial relationships that could be construed as a potential conflict of interest.

## Footnotes

1 http://blast.ncbi.nlm.nih.gov/Blast.cgi

2 http://rdp.cme.msu.edu/

## Supplementary Material

FIGURE S1 **Relative quantification of gene expression in bacteria-stimulated (A61 – *Pseudoalteromonas* sp., A55 – *Alteromonas* sp., L3 – *Lactobacillus casei*, A18 – *Enterovibrio coralii*) and unstimulated (control) peripheral blood leukocytes (PBL) of the European sea bass (*Dicentrarchus labrax*): **(A)** lysozyme; **(B)** Mx protein; **(C)** caspase 3; **(D)** tumor necrosis factor-α; **(E)** interleukine-10).** *X*-axis, sampling time-points post-stimulation; *Y*-axis, arbitrary units of gene expression; ^∗^, statistical significance at *p* < 0.05 level between fold change of stimulated vs. unstimulated PBL.

FIGURE S2 **Two-dimensional hierarchical clustering and heatmap display of genes grouped according to their expression profiles and bacterial isolates based on elicited responses.** Color key gives a range of colors for downregulated (-2 to 0, *Z*-score) and upregulated (0–2, *Z*-score) targets (IL10, interelukine-10; TNF, tumor necrosis factor-α; Casp3, caspase 3; LYS, lysozyme; Mx, Mx protein). Responses PBL of the European sea bass (*Dicentrarchus labrax*) in respect to time-points post-stimulation (1, 3, 5, and 12 h) and bacterial isolates (Pse – *Pseudoalteromonas* sp., Alt – *Alteromonas* sp., Lact – *Lactobacillus casei*, Ent – *Enterovibrio coralii*) are shown on bottom horizontal axes.

FIGURE S3 **(A)** Number of peripheral blood phagocytic leukocytes activated over four time-points (1, 3, 5, and 12 h) after stimulation by four bacterial isolates. **(B)** Respiratory burst of stimulated and non-stimulated (control) peripheral blood leukocytes by four bacterial isolates over four time-points (1, 3, 5, and 12 h) expressed as units of optical density (OD630).
