# Supplementary material for: Autochthonous Bacterial Isolates Successfully Stimulate In vitro Peripheral Blood Leukocytes of the European Sea Bass (Dicentrarchus labrax)
Source: Front Microbiol. 2016 Aug 8;7:1244. doi: 10.3389/fmicb.2016.01244 (PMC4976100; doi:10.3389/fmicb.2016.01244)
Supplement: Supplementary file 1 [file Table_1.PDF]

sample	BC id	GenBank id	RDP id	GenBank #
A2	*Vibrio*	***V. harveyi***/*V. rotiferianus*/*V. owensii*	***V. harveyi***	*KX356388*
A3	*Vibrio*	*Vibrio*	***V. harveyi***/*Vibrio* sp.	*KX356389*
A4	*Vibrio*	***V. coralliilyticus***/*Vibrio* sp.	***V. coralliilyticus***/*Vibrio* sp.	*KX356390*
A5	*Vibrio*	***Enterovibrio* sp.**/*E. coralii*	***Enterovibrio*** sp./*E. coralii*	*KX356391*
A6	*Aeromonas*	uncultured bacterium/ uncultured *Marinobacterium* sp.	uncultured bacterium/ uncultured *Marinobacterium* sp.	*KX356392*
A7	*Vibrio*	***Vibrio* sp.**/*V. sinaloensis*	***Vibrio* sp.**/*V. sinaloensis*	*KX356393*
A8	*Vibrio*	***Vibrio* sp.**/*V. sinaloensis*	***Vibrio* sp.**/*V. sinaloensis*	*KX356394*
A9	*Vibrio*	***V. harveyi***/*Vibrio* sp.	***V. harveyi***/*Vibrio* sp.	*KX356395*
A10	*Vibrio*	***Enterovibrio* sp.**/*E. coralii*	***Enterovibrio* sp.**/*E. coralii*	*KX356396*
A11	*Vibrio*	***Enterovibrio* sp.**/*E. coralii*	***Enterovibrio* sp.**/*E. coralii*	*KX356397*
A12	*Vibrio*	***Enterovibrio* sp.**/*E. coralii*	***Enterovibrio* sp.**/*E. coralii*	*KX356398*
A13	*Vibrio*	***Enterovibrio* sp.**/*E. coralii*	***Enterovibrio* sp.**/*E. coralii*	*KX356399*
A14	*Vibrio*	***Enterovibrio* sp.**/*E. coralii*	***Enterovibrio* sp.**/*E. coralii*	*KX356400*
A15	*Vibrio*	***Enterovibrio* sp.**/*E. coralii*	***Enterovibrio* sp.**/*E. coralii*	*KX356401*
A16	*Vibrio*	***Enterovibrio* sp.**/*E. coralii*	***Enterovibrio* sp.**/*E. coralii*	*KX356402*
A18	*Vibrio*	***Enterovibrio* sp.**/*E. coralii*	***Enterovibrio* sp.**/*E. coralii*	*KX356403*
A19	*Vibrio*	***Enterovibrio* sp.**/*E. coralii*	***Enterovibrio* sp.**/*E. coralii*	*KX356404*
A20	*Vibrio*	***Enterovibrio* sp.**/*E. coralii*	***Enterovibrio* sp.**/*E. coralii*	*KX356405*
A21	*Vibrio*	***V. harveyi***/*Vibrio* sp.	***V. harveyi***/*Vibrio* sp.	*KX356406*
A22	*Vibrio*	***V. sinaloensis***/*Vibrio* sp.	***V. sinaloensis***/*Vibrio* sp.	*KX356407*
A23	*Vibrio*	***Vibrio* sp.**/*V. coralliilyticus*	***Vibrio* sp.**/*V. coralliilyticus*	*KX356408*
A24	*Vibrio*	***V. harveyi*/*V. rotiferianus***	***V. harveyi*/*V. rotiferianus***	*KX356409*
A25	*Vibrio*	***Enterovibrio* sp.**/*E. coralii*	***Enterovibrio* sp.**/*E. coralii*	*KX356410*
A26	*Vibrio*	***Enterovibrio* sp.**/*E. coralii*	***Enterovibrio* sp.**/*E. coralii*	*KX356411*
A27	*Vibrio*	***V. harveyi***	***V. harveyi***	*KX356412*
A28	*Vibrio*	***V. rotiferianus***/*V. harveyi*	***V. rotiferianus***/*V. harveyi*	*KX356413*
A29	*Vibrio*	***Enterovibrio* sp.**/*E. coralii*	***Enterovibrio* sp.**/*E. coralii*	*KX356414*
A30	*Vibrio*	***Enterovibrio* sp.**/*E. coralii*	***Enterovibrio* sp.**/*E. coralii*	*KX356415*
A31	*Vibrio*	***Enterovibrio* sp.**/*E. coralii*	***Enterovibrio* sp.**/*E. coralii*	*KX356416*
A32	*Vibrio*	***V. harveyi***	***V. harveyi***	*KX356417*
A33	*Vibrio*	***V. harveyi***	***V. harveyi***	*KX356418*

A34	*Vibrio*	***Enterovibrio* sp.**/*E. coralii*	***Enterovibrio* sp.**/*E. coralii*	*KX356419*
A35	*Vibrio*	***V. mediterranei***	***V. mediterranei***	*KX356420*
A36	*Vibrio*	***Enterovibrio* sp.**/*E. coralii*	***Enterovibrio* sp.**/*E. coralii*	*KX356421*
A37	*Vibrio*	***Vibrio* sp.**/*V. sinaloensis*	***V. sinaloensis***	*KX356422*
A38	*Vibrio*	***Enterovibrio* sp.**/*E. coralii*	***Enterovibrio* sp.**/*E. coralii*	*KX356423*
A39	*Vibrio*	***V. harveyi***	***V. harveyi***	*KX356424*
A40	*Vibrio*	***V. harveyi***	***V. harveyi***	*KX356425*
A41	*Vibrio*	***Enterovibrio* sp.**/*E. coralii*	***Enterovibrio* sp.**/*E. coralii*	*KX356426*
A42	*Vibrio*	***Enterovibrio* sp.**/*E. coralii*	***Enterovibrio* sp.**/*E. coralii*	*KX356427*
A44	*Vibrio*	***Enterovibrio* sp.**/*E. coralii*	***Enterovibrio* sp.**/*E. coralii*	*KX356428*
A45	*Vibrio*	***Enterovibrio* sp.**/*E. coralii*	***Enterovibrio* sp.**/*E. coralii*	*KX356429*
A46	*Vibrio*	***Enterovibrio* sp.**/*E. coralii*	***Enterovibrio* sp.**/*E. coralii*	*KX356430*
A47	*Aeromonas*	uncultured bacterium/ uncultured *Marinobacterium* sp.	uncultured bacterium/ uncultured *Marinobacterium* sp.	*KX356431*
A48	*Vibrio*	***Vibrio* sp.**/*V. sinaloensis*	***Vibrio* sp.**/*V. sinaloensis*	*KX356432*
A49	*Vibrio*	***V. harveyi/V. rotiferianus***	***V. harveyi/V. rotiferianus***	*KX356433*
A50	*Vibrio*	***V. rotiferianus***/*V. harveyi*	***V. rotiferianus***/*V. harveyi*	*KX356434*
A51	*Aeromonas*	***Nautella* sp.**/*Nautella italic*	***Nautella italica***	*KX356435*
A52	*Vibrio*	***V. harveyi***	***V. harveyi***/*Aliivibrio fisheri*	*KX356436*
A53	*Vibrio*	***Vibrio* sp.**/*V. sinaloensis*	***Vibrio* sp.**/*V. sinaloensis*	*KX356437*
A54	*Vibrio*	***Enterovibrio* sp.**/*E. coralii*	***Enterovibrio* sp.**/*E. coralii*	*KX356438*
A55	*Aeromonas*	***Alteromonas macleodi*/ *Alteromonas* sp.**	***Alteromonas* sp.**	*KX356439*
A56	*Vibrio*	***V. harveyi***	***V. harveyi***/*Aliivibrio fisheri*	*KX356440*
A57	*Vibrio*	***V. owensii/V. harveyi/V. coralliilyticus***	***V. harveyi/V. coralliilyticus***	*KX356441*
A58	*Vibrio*	***V. harveyi***	***V. harveyi/Aliivibrio fisheri***	*KX356442*
A59	*Vibrio*	***Vibrio* sp.**/*V. sinaloensis*	***Vibrio* sp.**/*V. sinaloensis*	*KX356443*
A60	*Vibrio*	***Vibrio* sp.**/*V. sinaloensis*	***Vibrio* sp.**/*V. sinaloensis*	*KX356444*
A61	*Photobacterium*	***Pseudoalteromonas shioyasakiensis/P. lipolytica/ Pseudoalteromonas* sp./*P. nigrifaciens***	***Pseudoalteromonas* sp./*P. nigrifaciens*/ *P. lipolytica***	*KX356445*
A62	*Vibrio*	***Enterovibrio* sp.**/*E. coralii*	***Enterovibrio* sp.**/*E. coralii*	*KX356446*
A63	*Vibrio*	***Enterovibrio* sp.**/*E. coralii*	***Enterovibrio* sp.**/*E. coralii*	*KX356447*
A64	*Vibrio*	***Enterovibrio* sp.**/*E. coralii*	***Enterovibrio* sp.**/*E. coralii*	*KX356448*

A65	*Vibrio*	***Vibrio* sp.**/*V. harveyi*	***Vibrio* sp.**/*V. harveyi*	*KX356449*
A66	*Vibrio*	***Enterovibrio* sp.**/*E. coralii*	***Enterovibrio* sp.**/*E. coralii*	*KX356450*
A67	*Vibrio*	***Vibrio* sp.**/*V. sinaloensis*	***Vibrio* sp.**/*V. sinaloensis*	*KX356451*
A68	*Vibrio*	***Enterovibrio* sp.**/*E. coralii*	***Enterovibrio* sp.**/*E. coralii*	*KX356452*
A69	*Vibrio*	***V. harveyi/V. rotiferianus***/ ***Vibrio* sp.**	***V. harveyi/V. rotiferianus***/ ***Vibrio* sp.**	*KX356453*
A70	*Vibrio*	***Enterovibrio* sp.**/*E. coralii*	***Enterovibrio* sp.**/*E. coralii*	*KX356454*
A71	*Vibrio*	***Enterovibrio* sp.**/*E. coralii*	***Enterovibrio* sp.**/*E. coralii*	*KX356455*
A72	*Vibrio*	***V. rotiferianus***/*Vibrio* sp./*V. harveyi*	***V. rotiferianus***/*V. harveyi*	*KX356456*
A73	*Vibrio*	***Vibrio* sp.**/*V. sinaloensis*	***Vibrio* sp./*V. sinaloensis***	*KX356457*

Supplementary Table 1: Seventy isolates of autochthonous intestinal bacteria of the European sea bass (*Dicentrarchus labrax*) identified by biochemical keys (BC id) and molecular data blast of GenBank (GenBank id) and Ribosomal Data Base (RBD id). Identifications that corresponded both based on GenBank and RBD are in bold, while isolates used in experiments are highlighted in colors.

primer pair: forward and reverse (5'-3')	primers name	locus	~bp	annealing T_m (ºC)	use	reference
AGAGTTTGATCCTGGCTCAG, GGTTACCTTGTTACGACTT	27f, 1492r	16 small ribosomal RNA	1000	55	PCR	Wilson et al., 1960.
GGCATCACACACCATGG, CAGTGTGTCCACCTCC	LYSfDl, LYSrDl	lysozyme	200	52	qPCR	Buonocore et al., 2014
GGTCAAGGAGCAGATCAAACAG, CTCGCATCAGGTTAGGGAATC	MxfDl, MxrDl	Mx protein	130	57.7	qPCR	El Aamri et al., 2015
ACGAAGCAGGTCAATCATCC, GCAGTTTAAGGGTATCCAGAGC	Casp3fDl, Casp3rDl	caspase 3	98	59.3	qPCR	El Aamri et al., 2015
ACAGCGGATATGGACGGTG, GCCAAGCAAACAGCAGGAC	TNFfDl, TNFrDl	tumour necrosis factor α	76	60	qPCR	El Aamri et al., 2015
ACCCCGTTCGCTTGCCA, CA TCTGGTGACA TCACTC	IL10Dl, IL10Dl	interleukin 10	163	60	qPCR	El Aamri et al., 2015
CTGGTGTTGGTGAGTTCGAGG GGGGTTGTAGCCGATCTTCTTG	EF1Af1 EF1Ar1	elongation factor I α	203	60	qPCR	this study
TGCTGTCCCTGTATGCCTCTG GGCTGTGGTGGTGAAGGAGTAG	SBactinf2 SBactinr2	ß-actin	176	65	qPCR	this study
CCAACGAGCTGCTGACC CCGTTACCCGTGGTCC	18SFW 18SRV	18s rRNA	200	52	qPCR	Buonocore et al., 2014

Supplementary Table 2: Characteristic of primers for molecular identification of bacteria isolated from the European sea bass (*Dicentrarchus labrax*) intestine, and for relative quantification of target genes expression in bacteria-stimulated sea bass peripheral blood leukocytes (PBL).

groups	factor "Time"	P (MC)
Alteromonas sp. x *Pseudoalteromonas* sp.	1h	0.011
Alteromonas sp. x *Enterovibrio coralii*		0.03
Alteromonas sp. x *Lactobacillus casei*		0.048
Pseudoalteromonas sp. x *Lactobacillus casei*		0.028
Alteromonas sp. x *Enterovibrio coralii*	3h	0.041
Pseudoalteromonas sp. x *Enterovibrio coralii*		0.039
Enterovibrio coralii x *Lactobacillus casei*		0.049

Supplementary Table 3: Statistically significant difference in phagocytic activity of peripheral blood leukocytes of the European sea bass (*Dicentrarchus labrax*) stimulated by four bacterial isolates (groups) at 1 and 3 h (factor "Time") was evidenced by 3 way Permanova, with p-values obtained using 999 permutations of raw data with Monte-Carlo simulation (P (MC)).

groups	factor "Time"	P (MC)
Pseudoalteromonas sp. x control no-*Pseudoalteromonas* sp.	1h	0.012
Enterovibrio coralii x control no-*Enterovibrio coralii*		0.007
Lactobacillus casei x control no-*Lactobacillus casei*		0.003
Pseudoalteromonas sp. x control no-*Pseudoalteromonas* sp.	3h	0.035
Enterovibrio coralii x control no-*Enterovibrio coralii*		0.002
Lactobacillus casei x control no-*Lactobacillus casei*		0.004
Enterovibrio coralii x control no-*Enterovibrio coralii*	5h	0.003
Lactobacillus casei x control no-*Lactobacillus casei*		0.003
Enterovibrio coralii x control no-*Enterovibrio coralii*	12h	0.004

Supplementary Table 4: Statistically significant difference in respiratory burst of bacteria-stimulated and non-stimulated (control) peripheral blood leukocytes of the European sea bass (*Dicentrarchus labrax*) at 1, 3, 5 and 12 h (factor "Time") was evidenced by 3 way Permanova with p-values obtained using 999 permutations of raw data with Monte-Carlo simulation (P (MC)).
