# Supplementary figures and images for: Autochthonous Bacterial Isolates Successfully Stimulate In vitro Peripheral Blood Leukocytes of the European Sea Bass (Dicentrarchus labrax)
Source: Front Microbiol. 2016 Aug 8;7:1244. doi: 10.3389/fmicb.2016.01244 (PMC4976100; doi:10.3389/fmicb.2016.01244)

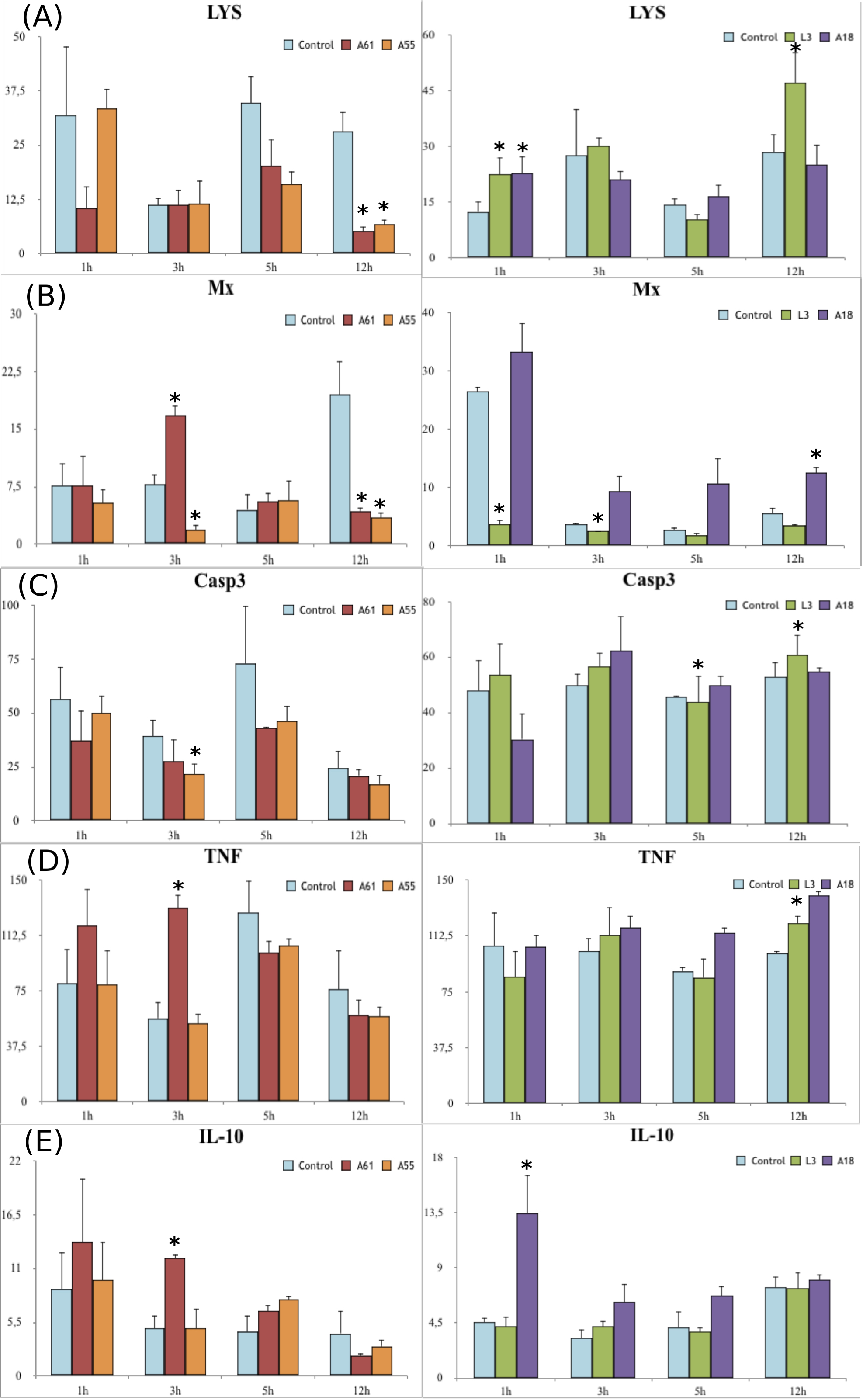

Supplement: FIGURE S1 — Relative quantification of gene expression in bacteria-stimulated (A61 – Pseudoalteromonas sp., A55 – Alteromonas sp., L3 – Lactobacillus casei, A18 – Enterovibrio coralii) and unstimulated (control) peripheral blood leukocytes (PBL) of the European sea bass (Dicentrarchus labrax): (A) lysozyme; (B) Mx protein; (C) caspase 3; (D) tumor necrosis factor-α; (E) interleukine-10). X-axis, sampling time-points post-stimulation; Y-axis, arbitrary units of gene expression; ∗, statistical significance at p < 0.05 level between fold change of stimulated vs. unstimulated PBL. [file Image_1.JPEG]

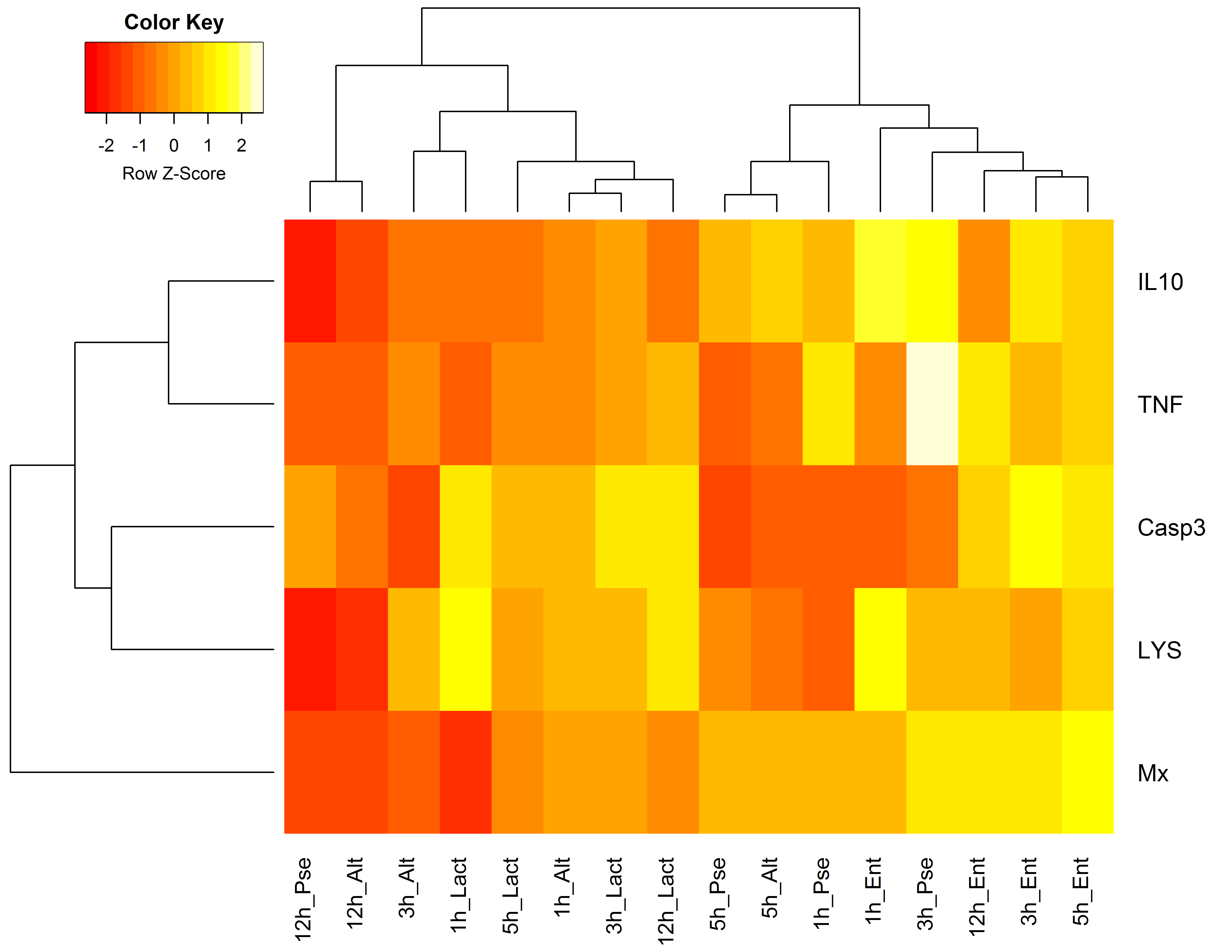

Supplement: FIGURE S2 — Two-dimensional hierarchical clustering and heatmap display of genes grouped according to their expression profiles and bacterial isolates based on elicited responses. Color key gives a range of colors for downregulated (-2 to 0, Z-score) and upregulated (0–2, Z-score) targets (IL10, interelukine-10; TNF, tumor necrosis factor-α; Casp3, caspase 3; LYS, lysozyme; Mx, Mx protein). Responses PBL of the European sea bass (Dicentrarchus labrax) in respect to time-points post-stimulation (1, 3, 5, and 12 h) and bacterial isolates (Pse – Pseudoalteromonas sp., Alt – Alteromonas sp., Lact – Lactobacillus casei, Ent – Enterovibrio coralii) are shown on bottom horizontal axes. [file Image_2.JPEG]

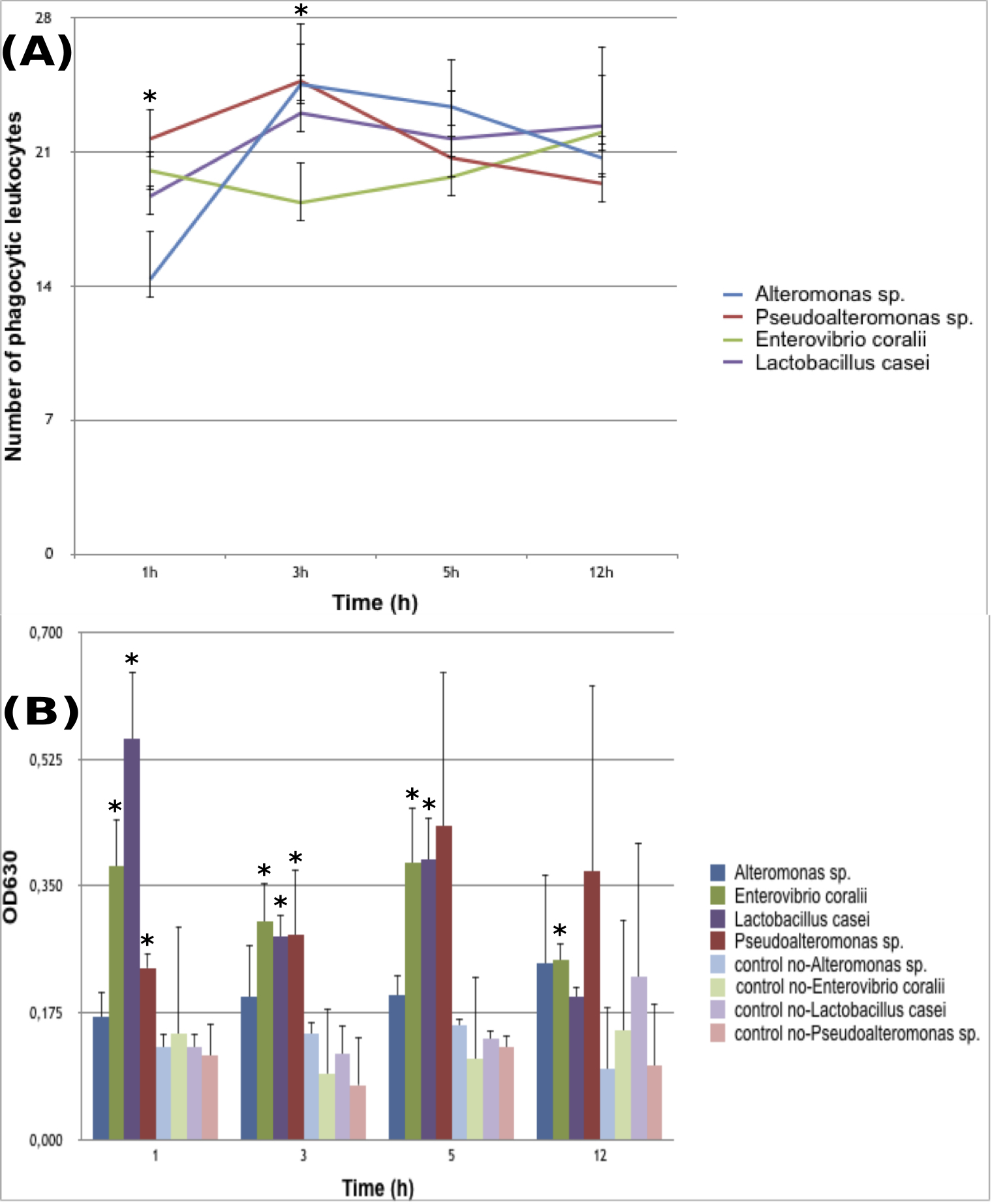

Supplement: FIGURE S3 — (A) Number of peripheral blood phagocytic leukocytes activated over four time-points (1, 3, 5, and 12 h) after stimulation by four bacterial isolates. (B) Respiratory burst of stimulated and non-stimulated (control) peripheral blood leukocytes by four bacterial isolates over four time-points (1, 3, 5, and 12 h) expressed as units of optical density (OD630). [file Image_3.JPEG]
